# Giant Primary Scrotal Lipoma: A Rare Entity with Diagnostic Pitfalls

**DOI:** 10.1155/2020/8815845

**Published:** 2020-11-05

**Authors:** Louis Vignot, Elie Saad, Michael Peyromaure, Nicolas Barry Delongchamps

**Affiliations:** Department of Urology, Cochin Hospital, University of Paris Descartes, Paris, France

## Abstract

Primary scrotal lipomas are rare. We describe the case of a 47-year-old male with a giant scrotal lipoma who underwent a surgical excision. We report the clinical and radiological approach as well as the treatment of this atypical benign tumor.

## 1. Introduction

In urological practice, intrascrotal lesions are frequent, and the major challenge is to distinguish a benign lesion from a malignant one. Lipomas represent a small proportion of scrotal swellings, but it is necessary to have knowledge of this benign entity as a differential diagnosis.

## 2. Case Presentation

The patient was a 46-year-old man with no relevant medical history who presented in our institution with a large left scrotal mass. He first noticed a swelling ten years ago and reported a very progressive growth of this lump that became discomfortable and dysesthetic. No other symptoms were reported in anamnesis. Physical examination revealed a large, nonreductible, soft, and painless left scrotal mass with well-defined margins. Both testicles were not palpable due to the large size of the mass.

**Figure 1:**
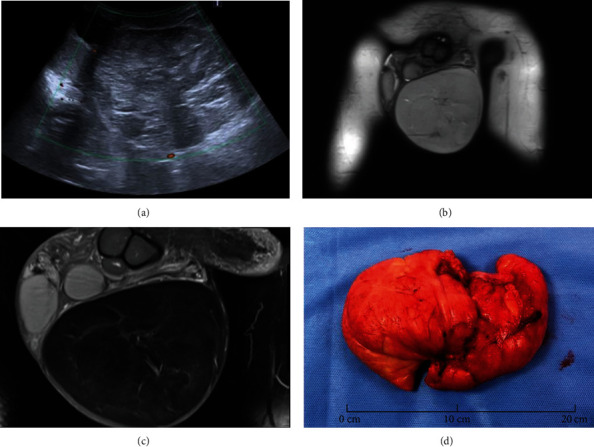
Imaging and gross pathologic features of primary scrotal lipoma. (a) On a color Doppler image, the lesion lacks internal flow. (b) Coronal T2-weighted image of the scrotum shows a lesion with homogeneous high signal intensity. (c) Coronal contrast-enhanced T1-weighted MRI shows very low enhancement of the tumor. (d) Gross examination of the surgical specimen.

The patient underwent scrotal ultrasonography (Figure 1(a)) to confirm the prima facie clinical diagnosis of hydrocele. The scrotal ultrasound revealed a large heterogeneous solid mass of 16 × 12 × 8 cm, showing weak vascularization on Doppler imaging, pushing both testicles upwards and to the right. The testicles, epididymis, and spermatic cords were intact.

Magnetic resonance imaging (MRI) showed a large extratesticular and extraepididymal mass with smooth margins and no intra-abdominal extension through the inguinal canal (Figures 1(b) and 1(c)). T2-weighted sequences revealed an encapsulated fat-containing mass, and T1-contrast enhanced sequences showed a very slight enhancement. This initial workup did not show any evidence of invasiveness of adjacent structures and did not support the diagnosis of malignant tumor. The most likely diagnosis was a lipoma. However, the radiologist could not formally rule out a diagnosis of liposarcoma because of the tumor's size.

An ultrasound guided biopsy was performed and confirmed the diagnosis of lipoma.

Surgical removal of the left scrotal mass was planned under general anesthesia, and a scrotal incision was performed. The lump appeared to be composed of fat, looked encapsulated, and was easily removed (Figure 1(d)). The testicles were not examined intraoperatively, because the tunica vaginalis testis was not opened and stayed intact during the whole procedure.

Immediate postoperative course was uneventful, and the patient was discharged on postoperative day one. Unfortunately, postoperative period was marked by the occurrence of a voluminous scrotal hematoma on day four, which required reintervention for drainage.

On gross examination, the specimen weighted 480 g, measured 17 × 11 × 6 cm and was roughly oval. It was multilobulated, yellowish with soft to firm consistency. Histopathological examination showed a proliferation of well-differentiated and mature adipocytes of various sizes in all samples.

## 3. Discussion

Intrascrotal lesions are a heterogeneous entity. Although benign in most cases, clinical examination alone may not exclude malignancy. Many diagnostic pitfalls exist, and multiple anatomical structures in a confined space with all their own pathologies do not make the clinician's task any easier [1].

Scrotal lipomas can originate from three different regions: (a) from the adipose tissue posterior to the spermatic cord, (b) from the spermatic cord, and (c) from the scrotal wall [2]. The last entity is called “primary scrotal lipoma” and is rarely seen. In most cases, it is not easy to identify the precise origin of the lesion.

Intratesticular masses are 95% malignant while nonepididymal extratesticular tumors, including those originating from the scrotum and spermatic cord, are most commonly mesenchymal benign tumors (97%) [3]. However, a diagnosis of extratesticular malignancy such as liposarcoma, leiomyosarcoma, lymphoma, or mesothelioma should not be missed. Although lipomas are the most common benign neoplasms in the scrotum, they remain a rare entity [4].

To our knowledge, there are very few cases of primary scrotal lipoma described in the scientific literature. Symptoms and patient discomfort vary according to the size of the lipoma: their weight varies from 225 g to 9 kg [5]. Clinically, it can mimic an inguinal hernia or a scrotal hematoma, hence the essential contribution of imaging. Ultrasonography is the first-line imaging and helps to specify the solid or cystic nature of the lesion as well as its location. While most of the extratesticular solid tumors are benign like the one reported, there may be some doubt especially concerning very large tumors. Magnetic resonance imaging (MRI) is then a valuable tool in such cases because it is the most precise resource in distinguishing a malignant tumor from a benign one. A homogeneous, encapsulated, and well-limited fat mass would be strongly in favor of a benign lipoma. But a huge tumor, although benign, may have a small proportion of muscle vessels or fibers, mimicking a well-differentiated liposarcoma. Moreover, O'Donnell et al. showed that experienced observers could differentiate well-differentiated liposarcoma from benign lipoma in only 69% of cases using only MRI [6]. Lipoma can be differentiated from liposarcoma by the lack of any enhancing soft tissue within a lipoma and more heterogeneous content of liposarcomas [3].

The biopsy performed in this context does not exclude a malignant focus because of the size of the lesion and its possible heterogeneity. Being an encapsulated and noninfiltrative mass helped us differentiate it from lipomatosis and the absence of abnormal mitosis and cellular atypia to differentiate from liposarcoma. Some very rare cases of malignant degeneration of giant scrotal lipomas have been described in scientific literature [7].

## 4. Conclusion

Primary scrotal lipomas are rare and benign tumors but should be kept in mind by the clinician as a differential diagnosis of scrotal swelling.

First line imaging remains ultrasound but does not allow differentiating a lipoma from a liposarcoma with certainty and must therefore be complemented by scrotal MRI, especially in the case of a large tumor.

The treatment of choice is surgical excision, and the histological analysis of the specimen allows a diagnosis of certainty.
